# Limb Length Discrepancy and Angular Deformity due to Benign Bone Tumors and Tumor-like Lesions

**DOI:** 10.5435/JAAOSGlobal-D-20-00214

**Published:** 2021-03-10

**Authors:** Taylor J. Reif, Julia Matthias, Austin T. Fragomen, S. Robert Rozbruch

**Affiliations:** From the Hospital for Special Surgery, New York, NY (Dr. Reif, Dr. Fragomen, and Dr. Rozbruch), and the Charité—Universitätsmedizin Berlin, Berlin, Germany (Ms. Matthias).

## Abstract

Benign bone tumors and tumor-like lesions are frequently diagnosed in children and adolescents. The immature skeleton is at risk for growth disturbances and deformity because of the effects of the lesions on normal bone architecture and the physis. The development, manifestation, and severity of the limb length inequality and deformity differs between the various bone pathologies. Distraction osteogenesis, osteotomy, and guided growth are key tools in the treatment of limb inequality and deformity using a combination of external and internal fixation devices.

Benign bone tumors and tumor-like lesions are bone abnormalities that create a wide spectrum of disease in human beings. Although some have the potential to transform into malignancy later in life,^1,2^ they are not life threatening at the time of diagnosis. However, many are deleterious to growth and skeletal maturation, inhibiting the physis or normal bone architecture leading to limb length discrepancy (LLD), deformity, or pathological fracture. Neglect of these lesions can also induce abnormal posturing, scoliosis, aberrant loading of joints, loss of function, and early degenerative changes.^3^ These lesions are therefore an ongoing threat to normal skeletal development. However, timely surgical intervention can improve patient outcomes.

Tumors are defined as masses with true neoplastic cells, whereas tumor-like lesions are developmental abnormalities within a bone, which in some cases can be traced back to genetic derangements. Both frequently present in children, adolescents, and young adults but can be diagnosed at any age.^4^ In this review, we summarize the impact of common benign bone tumors and tumor-like lesions on physeal function, discuss the development of limb length discrepancies and angular deformities, and outline effective treatment strategies.

## Bone Tumors

### Osteoid Osteoma

Osteoid osteomas are benign bone-forming tumors that are well circumscribed and commonly occur juxtacortically in the diaphyseal area of long bones.^5^ Their hallmark is localized nocturnal pain responsive to salicylates.^6^ Radiographically, they are recognized by a small radiolucent nidus with stippled central calcification surrounded by an area of cortical thickening and sclerosis.^7^ Similar appearing but larger (>2 cm) lesions, especially in the posterior spine, are more likely an osteoblastoma.^6^ Modern treatment uses percutaneous radiofrequency ablation with excellent results (Table 1).^8^

**Table 1:** Rates of Deformity, Leg Length Discrepancy, Common Treatment Strategies, and Outcomes for Benign Bone Tumors and Tumor-like Lesions

Etiology	Rate of Deformity	Deformity Treatment Strategies	Outcome
Osteoid osteoma^8,15^	• 10%-20% cause overgrowth of bone or deformity	• Radiofrequency ablation	• Success rate >90% • <5% recurrence
Enchondromatosis^24,34,36^	• Affected bones usually deformed • LLD 10-25 cm at maturity (untreated)	• Guided growth • Circular external fixation/motorized lengthening nails ◦ Often combined with elastic/static IM nails for long-term stability	• Case reports success with solitary enchondroma, insufficient for Ollier disease • Combined treatment effects: ◦ Mean treatment time 231 days ◦ Bone healing index (days/cm): femur 33.3; tibia 34.0 ◦ Complication rate 27.9% • Normal bone architecture often found after treatment
Multiple hereditary exostosis^2,38,40,46,48,51^	• 40% short stature (<20%tile) • 50% LLD in lower extremity • 25% coxa valga • 33% valgus knee • 50% valgus ankle • 40%-74% deformity in forearm ◦ Ulnar shortening ◦ Increased radial bow	• Excision • Guided growth • Fibular lengthening • Ulnar lengthening	• Usually for pain and can prevent radial head dislocation • Genu valgus correction 67% satisfactory, but slower rate versus idiopathic osteochondroma • Distal radial correction improvement in RAA, carpal slip, ulnar tilt • Can correct tibiotalar angle 0.37° per month, but recurrence more common than non-MHE correction after removal • Corrected instability in talus in 100% of ankles • Improves pronosupination, RAA, carpal slip, and prevents and treats radial head dislocation
Unicameral bone cyst^58,73,74^	• Impair growth in ∼10% of cases • Proximal humerus varus most common deformity	• Close observation until skeletal maturity • External fixation • Motorized lengthening nails	• Upper extremity shortening and deformity well tolerated • Acute compression of cyst via osteotomy, followed by distraction osteogenesis (7 day latency) achieved healing in 20/20 cysts • Two patients reached lengthening goal of 5/6 cm with no complication after skeletal maturity
Aneurysmal bone cyst^74,79,82,83^	• Growth impairment more likely in juxtaphyseal lesions (∼35% of ABCs) • Causes proximal humeral varus and limits abduction • Lower extremity deformity not described	• Mainstay of treatment is eradication of lesional tissue before limb reconstruction • Fibular strut grafting in proximal humerus • External fixation	• Recurrence rate 20%-30% • Healing of cyst in 19/20 patients, correction of neck shaft angle 106.6 → 136, 1 recurrence • Acute curettage followed by distraction osteogenesis improved function and healed cyst in 4/5 patients, 1 recurrence with poor outcome
Fibrous dysplasia^87,88,90,96^	• Rate of deformity dependent on the severity of bone involvement ◦ Polyostotic FD—60% LLD • Shepard's crook is characteristic deformity of femur, 35% of PFD	• Bisphosphonates for pain and to prevent deformity • Closing wedge valgus osteotomy • Circular external fixation/motorized lengthening nails ◦ Often combined with elastic/static IM nails for long-term stability	• 80%-100% pain relief, thickened cortices, McCune-Albright less responsive • Neck shaft angle corrected from 88.1 to 128.5 in 10 patients. Plate and IM nail constructs effective. • Limited case series, short (∼4 cm) lengthening possible, dysplastic bone remains, avoid lesions if possible

ABC = aneurysmal bone cyst, FD, fibrous dysplasia; IM = intramedullary, LLD = limb length discrepancy, MHE = multiple hereditary exostosis, PFD = polyostotic fibrous dysplasia, RAA = radial articular angle

Although their peak frequency is in adolescence, most osteoid osteomas do not directly interact with the physis because of their remote localization.^9^ Nevertheless, a subgroup of intra-articular osteoid osteomas present with joint swelling, painful limitation of joint motion, synovial inflammation, and osseous deformity. An osteoid osteoma in the femoral neck for example can lead to an enlarged femoral head, coxa magna and valga, and femoral anteversion.^9,10^ Although these bone alterations were initially believed to develop secondary to muscle contractures and hyperemia because of delayed diagnosis,^11^ more recent case reports show the development of osseous abnormalities within a few months after the onset of symptoms and before the development of contractures, suggesting stimulation of osseous changes by the tumor itself.^10^ Detectable changes in morphology can be identified on CT or MRI in the articular surface and both sides of a joint.^12^ Local hyperemia and expression of cyclooxygenase-2 have been postulated as potential mechanisms.^13^ However, inflammation-induced physeal overgrowth with premature closure of the physis—as can be found in pediatric patients with arthritic inflammation of a different origin—is not found in osteoid osteoma–induced synovitis.^14^

Norman and Dorfman^9^ reviewed 64 osteoid osteomas and found 6, mostly located in the metaphysis, had overgrowth, and/or had deformity of the affected bone. After excision, the malalignment and the LLD regressed but did not always normalize, and bone overgrowth persisted. Peyser et al^8^ reported that 4/22 pediatric patients with osteoid osteoma had ipsilateral overgrowth of 10 to 20 mm that regressed after ablation of the tumor but did not specify the location of the tumor in the bone. A small risk of recurrence (<5%) after radiofrequency ablation exists, and many discrepancies regress with time; therefore, delayed treatment of residual LLD and deformity is acceptable.^9,15^ Several case reports exist of epiphyseal osteoid osteomas that did not cause growth disturbance despite being near the physis and were surgically removed without growth disruption.^16,17,18,19^ Thus, a minority of osteoid osteomas can cause bone overgrowth leading to LLD; however, this does not seem to be mediated by the physis in most cases.

### Enchondroma and Enchondromatosis

Enchondromas are benign ectopic remnants of hyaline cartilage usually found in the metaphyseal region of tubular bones.^20^ The etiology is a generalized disturbance of endochondral ossification because of abnormal maturation or resorption of physeal chondroblasts.^21^ Solitary enchondromas are common and usually present incidentally. Multiple enchondromatosis, known as Ollier disease, is a nonhereditary skeletal disorder in which multiple enchondromas occur with an asymmetrical distribution and a unilateral predominance.^20^ Radiographically, multiple nests of lesions are oriented along the longitudinal bone axis and seem as radiolucent, homogenous areas that carve out normal boney architecture, often with radiodense margins indicating their slow growth.^22^ Patients most commonly present with painless bulging masses on the phalanges and metacarpals, although long bones such as the femur and tibia are often affected.^22^

The presentation of Ollier disease is usually early childhood because multiple enchondromas near the physes cause severe progressive growth inhibition and angular deformity. The diaphysis of the affected bones is short and seems expanded, given normal radial growth amplified by protuberant tumor masses. Growth disturbance can be multifactorial, including erosion of the adjacent physis, tethering of the physis by bridging tumor, or abnormally thick periosteal sleeves formed in reaction to the tumor.^22^ Enchondromas, in particular, tend to cause a varus deformity of the knee because of medial distal femur tumors,^23,24^ although the underlying predilection for this location is not understood.

Traditional therapies for Ollier disease are similar to other benign entities including tumor excision by curettage, osteotomies for alignment correction along with bone grafting, and internal fixation for stabilization.^25^ However, these methods do not address the often severe limb length discrepancies.^26,27^ An attempt to untether the physis and stimulate native growth via excision of any bridging tumor is a logical first step in treatment. This was successfully used by Niethard et al,^24^ who achieved normal growth and regression of deformities after excising juxtaphyseal enchondroma tissue and applying a plate to the opposite healthy part of the physis, also known as guided growth. This approach is feasible if carried out early in life, with sufficient healthy physis remaining after tumor excision to support growth and enough time for deformity correction to occur.

**Figure 1:**
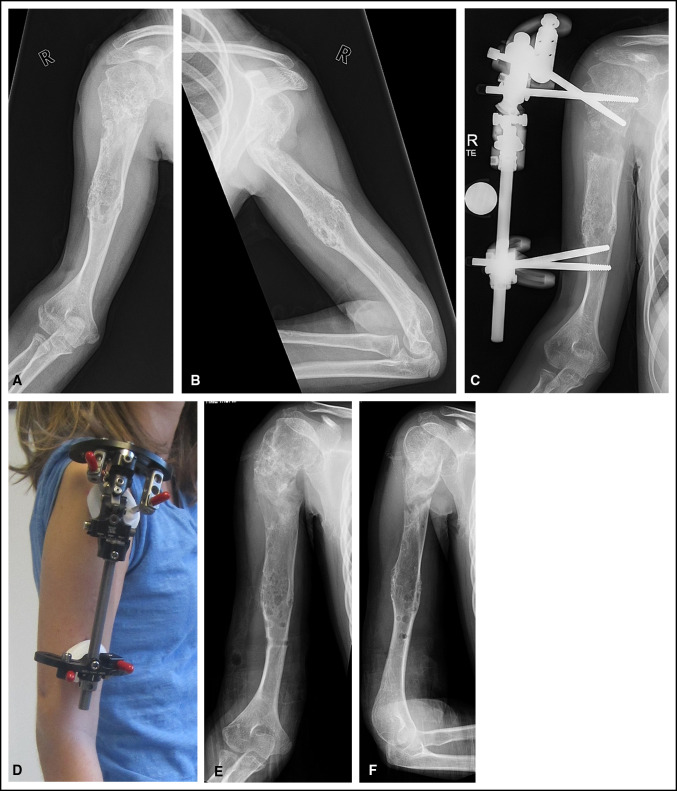
**A**, Figure demonstrating a patient with Ollier disease who underwent one humeral lengthening 5 years ago but has residual limb length discrepancy and limited growth potential remaining. **B**, Concurrent proximal humeral procurvatum deformity present. **C** and **D**, A monolateral rail was used to lengthen the bone and correct the deformity. **E** and **F**, After consolidation of new bone the rail is removed, and the deformity has been corrected and humerus lengthened.

The magnitude of LLD is often too large to correct with guided growth alone, so limb lengthening and angular correction by external fixator-assisted distraction osteogenesis has therefore been used in many cases.^28^ The chondral tissue invading the bone matrix is a special consideration in these patients because the cartilaginous matrix does not provide the same stability to half pin and wire fixation. Distraction osteogenesis will fail without adequate stabilization protecting the bone regenerate during and after the lengthening process. Baumgart et al^29^ presented a patient who experienced premature removals of several fixators because of the lack of stabilization. Several studies support the use of additional fixation points through normal and diseased bone to improve stability.^30,31^ Alternatively, in a series of nine patients, Popkov et al used a combination of external fixation paired with hydroxyapatite-coated intramedullary elastic nails to improve stability. The nails were left in place after lengthening to reduce the time in the fixator and its related complications.^32,33^ The lengthening over a nail technique uses the versatility of external fixator–driven lengthening and deformity correction while improving stability in the diseased bone. Given the severity of shortening in patients with Ollier disease, several repetitions of distraction osteogenesis may be required if growth was inhibited from a young age^34^ (Figure 1).

Another consideration regarding limb lengthening in patients with Ollier disease is the choice of osteotomy site. For correction of deformity without translation, the osteotomy is often made through the apex of deformity, also known as the center of rotation of angular deformity.^35^ This is often at the tumor site, and distraction must be conducted through the pathologic tissue. Enchondromas in Ollier disease carry a 30% to 40% risk of transforming into a malignant chondrosarcoma over the patient's lifetime (and up to 100% in Maffucci syndrome)^1^; hence, lengthening through tumor is controversial because it stimulates tissue differentiation. However, the risk so far seems theoretical because no reports exist of distraction leading to malignant transformation in Ollier disease. Several studies have observed the opposite in fact—conversion of chondral tumor tissue into normal bone during lengthening.^25,26,30,36^ This improves the overall stability of the bone by the increasing normal bone architecture. This phenomenon is likely related to the same tensile strain that promotes osteoblasts to create bone through intramembranous-like ossification during normal distraction osteogenesis and creating an unfavorable local milieu for ongoing chondrocyte maturation.^37^ Notably, Watanabe et al^31^ witnessed conversion of cartilaginous tissue to normal bone in only one of seven intralesional osteotomies; hence, distraction alone may not be sufficient in all cases, and further research is needed to understand this effect. Osteotomy should not be done if the patient is at high risk for malignant transformation (older age and previous malignancy) because identifying concerning radiographic changes will be more difficult after surgical intervention.

### Osteochondroma and Multiple Hereditary Exostosis

Osteochondromas are cartilage capped osseous tumors arising from progenitor cells in the inner layer of the perichondrium adjacent to the physis of tubular long bones.^2^ The medullary cavity is always confluent between the osteochondroma and normal bone. Solitary osteochondromas usually grow away from the adjacent joint, leading to a palpable mass and mechanical symptoms, but no disturbance of growth. Multiple hereditary exostosis (MHE) is an autosomal-dominant hereditary disorder characterized by multiple osteochondromas throughout the upper and lower extremities. MHE is diagnosed at a median age of three years old because of protuberant masses in the extremities.^38^ Osteochondromas associated with MHE are more likely to negatively affect the adjacent physis because of loss of function of the EXT1 or EXT2 proteins involved in heparan sulfate signaling pathways in the physis.^39^ Studies have shown a 8 to 10% prevalence of LLD and 25% of MHE patients in the 10 percentile or less for height.^40^ Porter et al^41^ via a radiographic analysis of the forearm demonstrated that the relative length of the bones correlates inversely with the relative size of the exostoses. Limb angulation is caused by growth differential between adjacent bones. Lack of fibular growth leads to knee and ankle valgus, whereas deficient ulnar growth leads to ulnar translocation of the carpus with increased radial bow. Denduluri et al^42^ also described three patients who experienced overgrowth of the medial proximal tibial physis, leading to genu valgum after surgical excision of a symptomatic osteochondroma, which they postulate may be related to hyperemia to the physis postsurgery.

**Figure 2:**
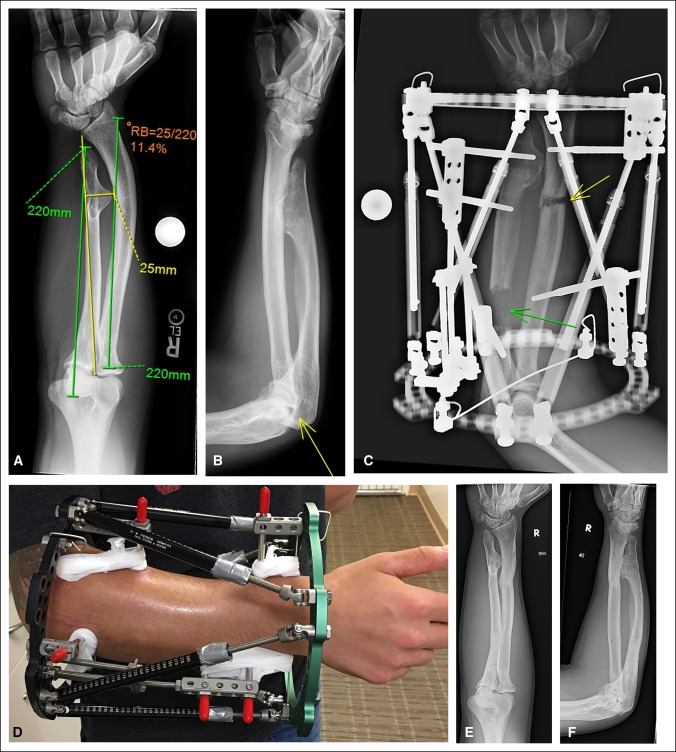
**A**, Figure demonstrating a patient with multiple hereditary exostosis who presented with gross deformity of forearm. The bone lengths are equal, but the radial bow is increased, leading to ulnar translocation of carpus (measurements originally described by Burgess et al). **B**, Although the radial head did not dislocate during growth in this patient, subluxation is evident (yellow arrow). **C** and **D**, External fixator used to lengthen the ulna (green arrow) and correct the radial deformity (yellow arrow). **E** and **F**, Radiographs of forearm after fixator removal demonstrating improved position of carpus and radial head.

MHE patients frequently have osteochondromas of the forearm, with 86% overall and 60 to 80% affecting the distal radius and ulna.^43^ The smaller distal ulnar physis commonly fails to keep pace with the radius, leading to ulnar shortening, bowing of the radius, and ultimately dislocation of the radial head. Radial head dislocation is best avoided because of functional restriction of the forearm which in one study resulted in a 36° loss of pronosupination.^43^ Relative shortening of the ulna also results in ulnar translocation of the carpus, increasing the ulnar-sided pressure on the radial epiphysis, causing angular deviation of the radial articular angle (RAA). Therapeutic approaches focus on osteochondroma excision, ulnar lengthening, and correction of the RAA to prevent or treat radial head dislocation and improve hand and wrist mechanics.^44^ The ideal time point for these interventions has been a matter of controversy. Treatment must weigh the possible need for multiple interventions because growth inhibition may remain after lengthening in young children, against younger patients' high remodeling potential which can correct deformity with further growth and prevent radial head dislocation. In a study of seven patients, Belyea et al^45^ found that osteochondroma excision alone with release of the ulnar-sided tether (triangular fibrocartilaginous complex) improved the carpal slip and prevented progression toward radial head dislocation, despite the perpetually increased RAA and relative ulnar shortening. However, Jo et al in a study of 102 limbs found that radial head dislocation had a positive correlation with ulnar length <0.9 the radius or radial bowing greater than 8.1%.^46,47^ Hence, although osteochondroma excision and tether release may be enough in certain patients, those with more severe ulnar shortening or radial bowing should be evaluated for ulnar lengthening to reduce radial head dislocation risk. Lengthening also improves the aesthetic appearance of the forearm and hand (Figure 2).

If the radial head is already dislocated, ulnar lengthening is still a good treatment option. Hsu et al^48^ compared the clinical outcome of children less than 10 treated with ulnar lengthening and children more than 10 treated with ulnar lengthening along with surgical correction of the RAA. They found that early lengthening restored the function of the forearm, improved the RAA, and led to spontaneous reduction of the dislocated radial head, with comparable outcomes with the older children without the need for additional corrective procedures of the distal radius. Cho and Jung^49^ reported spontaneous reduction of the radial head after gradual lengthening of the ulna using an Ilizarov external fixator without additional surgical interventions.

Similar to the distal ulna, osteochondromas often affect the growth of the distal fibular physis, leading to ankle valgus deformity, translational talus instability, and eventual degeneration of the ankle joint. The approach is similar to the forearm—excise the osteochondroma and lengthen the fibula by external fixation–assisted distraction-osteogenesis.^50^ Lee et al^51^ lengthened 12 fibulas in nine patients an average of 15.3 mm using a monolateral fixator with normalization of the medial talar clear space and correction of gross instability of the ankle.

**Figure 3:**
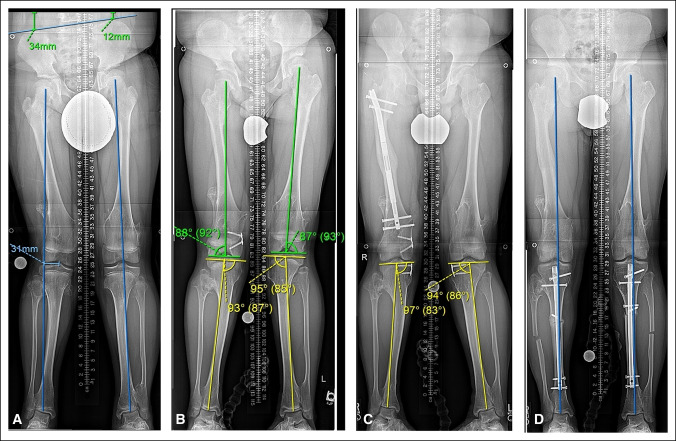
**A**, Figure demonstrating a patient with multiple hereditary exostosis presented with right leg length discrepancy of 22 mm and distal femur valgus deformity. **B**, Guided growth was successful in treating the distal femoral valgus, but bilateral proximal tibia valgus developed demonstrated by medial proximal tibia angle >90°. **C**, The proximal tibial valgus persisted despite 18 months of guided growth. A lengthening of the femur to correct the limb length discrepancy was also done during this time. **D**, Bilateral tibial osteotomies were used to correct the tibial valgus. Note the blocking screws proximal to the osteotomies to guide the path of the nails. Mechanical axis lines now pass through the center of the knees.

MHE also leads to osteochondroma formation around the knee in 90% of patients, with 70% of patients with at least one in the distal femur—the most common location (∼70%). Presence of a distal femur osteochondroma has been found to be a predictor of knee deformity, most commonly genu valgum (20% of patients) and procurvatum (16%), as well as short stature, likely related to the prominent role of the distal femoral physis in longitudinal growth (Figure 3).^40^ Proximal fibula osteochondroma–related growth arrest also contributes to genu valgum deformity. Ofiram et al^52^ used an Ilizarov fixator to accurately correct ankle and knee valgus in seven limbs with MHE, five of which had proximal and distal deformity requiring bifocal osteotomy and three of which underwent concurrent limb lengthening.

## Tumor-like Lesions

### Unicameral Bone Cysts

Unicameral bone cysts (UBCs) are common resorptive lesions of the bone which are found in the metaphyseal region of long bones, with a 2:1 male predominance.^53^ The proximal humerus is most commonly affected, accounting for 60% of UBCs, followed by the proximal femur.^54^ UBCs consist of a solitary fluid-filled cavity lined by a fibrous cyst membrane and present clinically with mild ache or pathological fracture.^55^ The cysts usually develop near the epiphyseal plate in young or adolescent patients; hence, the immature skeleton is at risk for growth impediment and deformity.

UBCs typically involute with skeletal maturity; hence, treatment is aimed at minimizing morbidity until that time. Some cysts will heal spontaneously after fracture and no intervention. The range of treatment options includes close observation and activity modification, minimally invasive procedures such as corticosteroid instillation, or surgical evacuation of the cyst by curettage, usually filling the defect with some combination of bone graft, demineralized bone matrix, calcium phosphate or sulfate, or hydroxyapatite.^55-57^ The choice of intervention must weigh the conflicting priorities of minimizing repetitive fractures by surgically stabilizing fragile cystic bone enabling young patients to partake in normal childhood activities and securing the growth potential of the physis which can be adversely affected by repeated or aggressive curettage.

Impaired growth of the affected limb is observed in approximately 10% of the cases.^58^ Patients can develop a LLD without experiencing a pathologic fracture,^59^ suggesting a direct negative impact of the cyst on the functionality of the physis, possibly mediated by an increased pressure within the cyst and local erosions of the physis.^60,61^ However, iatrogenic physeal injury during surgical interventions and repetitive fractures through the cystic lesions adjacent to the physis are frequently discussed as other causes of growth retardation.^62,63^ Neer et al^64^ showed a reduced frequency of growth impairment after surgical intervention compared with conservative treatment choices, suggesting that decompressing the cystic lesion and preventing frequent refractures outweigh the risk of iatrogenic physeal injury. However, fractures due to solitary bone cysts tend to affect the cortex distal to the physis, possibly protecting it from accompanying injury.^65^ Although rare, solitary bone cysts that progress through the open physis and expand into the epiphyseal region carry a particularly high risk of impairing growth.^61,66^ MRI evidence suggests the epiphyseal cysts develop via central erosions of the physis by a juxtaphyseal metaphyseal cyst.^61^ In the humerus, this is usually followed by a medial epiphyseal slip into varus and a ballooning of the epiphysis.^66^ Growth inhibition is likely caused by a combination of the central erosion and slipped physis.

**Figure 4:**
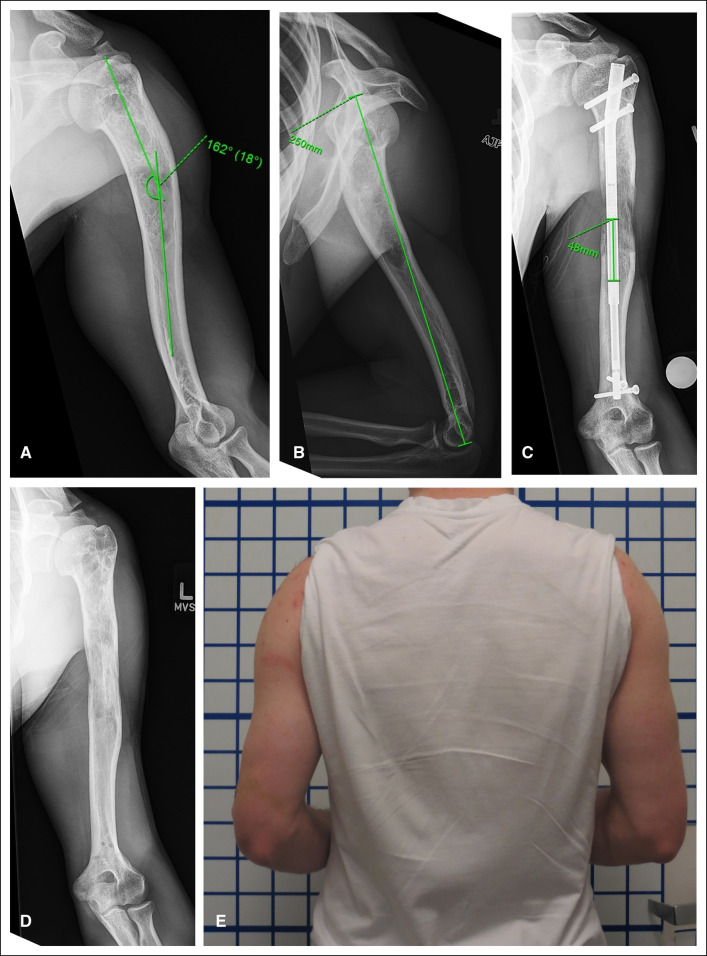
**A**, Figure demonstrating a patient with a history of proximal humerus unicameral bone cyst, now involuted with residual varus deformity. **B**, The humerus is short versus the contralateral which measured 300 mm; also with procurvatum deformity. **C**, An internal lengthening humeral nail was used to correct the deformity and lengthen the humerus; the nail has been distracted 48 mm. **D**, After consolidation of the new bone the nail is removed and the deformity has been corrected. **E**, The arm lengths have been equalized

Because the principle function of the upper extremity is to position the hand in space rather than bearing weight, small arm length discrepancies, up to 5 to 6 cm, can usually be tolerated without notable limitations in function.^67,68^ When the arm length discrepancy impairs the synergy of both arms or compromises the patient aesthetically because of severe asymmetry, lengthening should be considered.^69^ Lengthening is usually undertaken after skeletal maturity and cyst involution but can proceed earlier if a large adult discrepancy is anticipated. Distraction osteogenesis with external fixation^67,70-72^ and internal lengthening nails^73^ have led to satisfying results with a lower complication rate than lower extremity lengthening (Figure 4).

Some authors have recommended an alternative approach, using a pathologic fracture as an opportunity for distraction osteogenesis. Verdiyev and Verdiyev achieved cyst healing in 24 of 25 patients (20 UBCs and 5 aneurysmal bone cysts [ABCs]) when acute compression, followed by distraction osteogenesis was done through either the pathological fracture or an osteotomy at the distal end of the cyst. The distraction supports cyst decompression and stimulates new bone growth in the cavity. The benefit of this approach includes concurrent correction of angular deformities and limb length discrepancies along with definite cyst treatment. The treatment can take 4 to 6 months in an external fixator,^74^ but this approach may be warranted when a large cyst has a high risk of recurrent fracture (leading to recurrent periods of immobility), growth arrest, and deformity.

### Aneurysmal Bone Cysts

ABCs are rare bone lesions that cause extensive bone destruction and can lead to pathologic fracture or bone defect if left untreated.^75^ They tend to arise in the metaphyseal region of long bones such as the humerus, femur, or tibia. As opposed to most other bone tumors, ABCs slightly predominate in women.^76^ The primary appearance in growing adolescents combined with the aggressive and destructive behavior near the open physis presents a threat to growth of affected limbs. They also present secondary to other etiologies, such as giant cell tumor, chondroblastoma, and fibrous dysplasia; hence, careful radiographic and pathologic review is recommended.^77^

ABCs have reported recurrence rates up to 20% to 30%^78,79^; hence, the central goal of treatment is the complete removal of pathologic tissue. The approach includes thorough curettage and burring of the cavity to eliminate all crypts; adjuvant local treatments including phenol, liquid nitrogen, or argon beam coagulation; and grafting of the void with bone graft or ceramic substitute. Sclerotherapy, selective arterial embolization, doxycycline foam injection, and denosumab are alternative or adjuvant treatment strategies used in anatomically challenging areas such as the pelvis or spine.^80,81^ However, none of these therapies treat malalignment or limb shortening.

Causes of growth cessation are thought to be a direct result of erosion of the epiphyseal plate by the ABC or pathologic fracture adjacent to the physis.^82^ Especially large and juxtaphyseal bone cysts broaching more than two thirds of the proximal humeral physis are associated with limb shortening, varus angulation, and impaired shoulder abduction.^82^ Juxtaphyseal ABCs have a higher recurrence rate, 8 of 19 (42%) in one series, likely because of incomplete excision because of cautious surgical evacuation in these delicate areas.^75,79^ Despite this, Rizzo et al ^83^ still advocate for an intralesional excision of juxtaphyseal ABCs, demonstrating zero growth arrests in 15 patients. Mostafa and Fawzy^82^ demonstrated curettage and intramedullary fibular strut grafting seems to be a viable method to provide immediate mechanical support and alignment correction without the need for internal fixation devices. Good results were obtained in 18 of 20 patients using this technique; however, the treatment did not address limb length inequalities.

To address the limb shortening and restore the function of the upper extremity, Acan et al ^84^ successfully did distraction osteogenesis using a intramedullary lengthening nail in a 13-year-old boy with 5 cm humeral shortening caused by an aggressive recurrent ABC of the proximal humerus. Lengthening was done in the diaphyseal region distal to the bone cyst that was curetted but not grafted at the time of nail insertion. The bone cyst seemed totally healed at the end of the treatment. Similar to UBCs, Verdiyev and Verdiyev^74^ found complete healing of five ABCs after open curettage and distraction osteogenesis using external fixation. The simultaneous treatment of the cyst and LLD/deformity shortens the overall treatment time; however, given the not insignificant risk of recurrence, staged excision of the lesion followed by limb reconstruction may be prudent if the necessary fixation crosses the excised lesion.^79^

A dearth of literature exists on treatment of lower limb length discrepancies and malalignment due to ABCs. This may be because of the aggressiveness of these lesions that become symptomatic before notable growth arrest occurs or the subtle discrepancies caused are not recognized until adulthood. Regardless, future large series of lower extremity ABCs could shed valuable light on this topic.

### Fibrous Dysplasia

Fibrous dysplasia is a disorder of bone maturation into lamellar bone, resulting in lesions of dysplastic fibrous tissue containing abnormally shaped trabeculae of the woven bone.^85^ The lesions are commonly found in the diaphysis or metaphysis of long bones, especially the proximal femur.^86^ The disease is divided into a monostotic form, which has a higher incidence and is less severe or even asymptomatic, and a polyostotic form, which is associated with McCune-Albright syndrome (café au lait spots and multiple endocrine abnormalities) and Mazabraud syndrome (intramuscular myxomas) and carries an increased risk of pathologic fracture and malignant transformation.^87^ Spine radiographs to evaluate for scoliosis and a bone scan to determine extent of disease are recommended.^88^

The monostotic lesions usually enlarge in proportion to skeletal growth, whereas polyostotic lesions continue to enlarge after skeletal maturation.^87^ The bone affected by fibrous dysplasia is weak and prone to pathological fracture. The fractures often occur insidiously as microfractures with spontaneous healing but progressive angular deformity. This pattern in the proximal femur leads to progressive varus of the femoral neck and lateral cortical bending, ultimately appearing like a shepherd's crook.^89,90^ The shepherd's crook deformity is notoriously challenging to correct; reports of successful surgical intervention include a closing wedge valgus osteotomy stabilized with a fixed angle implant (nail or plate) along with cortical allograft to fill the defect that resorbs less readily than autograft.^91^

Growth arrest of the physis is not typical in fibrous dysplasia. In cases of epiphyseal fibrous dysplasia, disruption of physeal growth has not been noted, despite being near the open physis, even with extension into the metaphysis.^85,92-94^ Thus, limb length discrepancies in fibrous dysplasia stem from the angular deformity and fracture shortening rather than a lack of longitudinal growth.

**Figure 5:**
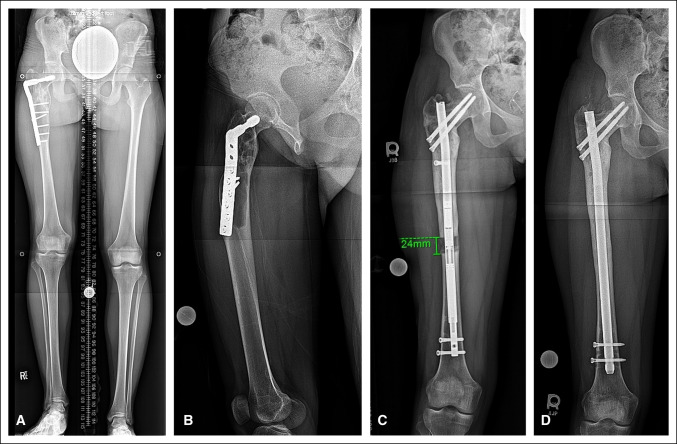
**A**, Figure demonstrating a patient with history of fibrous dysplasia and right proximal femur stabilization presented with pain in right hip and knee along with 1 inch leg length discrepancy (block under right foot). **B**, Despite previous surgery as a child, the fibrous dysplasia lesion is at ongoing risk of pathologic fracture. **C**, The lesion was bypassed and stabilized with an internal lengthening nail which corrected the limb length discrepancy via distraction osteogenesis. **D**, The lengthening nail was exchanged for a static locked nail to provide ongoing support of the pathologic bone.

Fibrous dysplasia lesions and associated deformities are difficult to treat surgically because of the tendency for the lesions to recur and resist healing with stable lamellar bone. Stanton et al^86^ reported formation of unstable dysplastic bone in the regenerate tissue during a lengthening procedure and recommended doing lengthening through healthy bone segments, which are scarce in patients with polyostotic fibrous dysplasia. Nonsurgical treatment with intravenous bisphosphonates is effective at relieving pain and improving bone quality, and more recently denosumab has also been shown to relieve bone pain refractory to bisphosphonates but needs more study before widespread use.^95^ When deformity is already present and surgery deemed necessary, Popkov et al^32^ combined external-fixator-assisted bone lengthening with intramedullary hydroxyapatite-coated elastic nails to increase the stability of the weak dysplastic bone. By leaving the nails in the lengthened bone, the dysplastic bone has support beyond the treatment time in the external fixator. Limb lengthening and deformity correction using external fixation or magnetic lengthening nails (Figure 5) has also been done successfully^70,96^; however, it is not always reported whether this was done through diseased or healthy bone segments. Notably, in a case report by Harris et al^97^ of distraction osteogenesis through a biopsy-proven distal femur fibrous dysplasia lesion, there arose an osteosarcoma in the regenerate region. Although rare, extended follow-up of any patient with benign disease who undergoes a lengthening procedure is recommended.

## Summary

Benign bone tumors and tumor-like lesions are commonly diagnosed in young patients with open physes and therefore have the potential to affect growth and cause deformity. Although the causes and manifestations of the related bony deformities are diverse, treatment concepts show similarities. Local control of the tumor/lesion is important but often insufficient—many patients will require some form of deformity correction or limb lengthening. Surgical techniques include osteotomy, distraction osteogenesis, epiphyseal plate modulation, and epiphysiodesis. The tools available are plates, screws, static and elastic intramedullary nails, lengthening intramedullary nails, and external fixators. Principles of limb lengthening and deformity correction help guide decision-making. External fixation is a versatile tool for limb lengthening and gradual deformity correction and can be combined with internal fixation techniques when ongoing support of pathologic bone is desired. Motorized internal lengthening nails are another powerful tool that will continue to grow in use as more surgeons become familiar with their capabilities.
